# Association between social disadvantage and falls in older adults: a longitudinal study

**DOI:** 10.15649/cuidarte.5220

**Published:** 2026-04-15

**Authors:** Juan David Salcedo Salgado, Igor Cigarroa, Rafael Tuesca, Salim Touchie, Tania Acosta

**Affiliations:** 1 Universidad de Magdalena, Santa Marta, Colombia. E-mail: Jsalcedos@unimagdalena.edu.co Universidad de Magdalena Santa Marta Colombia Jsalcedos@unimagdalena.edu.co; 2 Escuela de Kinesiología, Facultad de Ciencias de la Salud, Universidad Católica Silva Henríquez, Santiago, Chile. E-mail: icigarroac@ucsh.cl Universidad Católica Silva Henríquez Santiago Chile icigarroac@ucsh.cl; 3 Universidad del Norte, Departamento de salud pública, Barranquilla, Colombia. E-mail: rtuesca@uninorte.edu.co Universidad del Norte Barranquilla Colombia rtuesca@uninorte.edu.co; 4 Universidad Cooperativa de Colombia. Santa Marta, Colombia. E-mail: salin.touchie@ucc.edu.co Universidad Cooperativa de Colombia Santa Marta Colombia salin.touchie@ucc.edu.co; 5 Universidad del Norte, Departamento de salud pública, Barranquilla, Colombia. E-mail: tacosta@uninorte.edu.co Universidad del Norte Barranquilla Colombia tacosta@uninorte.edu.co

**Keywords:** Accidental Falls, Aged, Socioeconomic Disparities in Health, Occupations, Accidentes por Caídas, Anciano, Desventaja Social en Salud, Ocupaciones, Acidentes por Quedas, Idoso, Disparidades Socioeconômicas em Saúde, Ocupações

## Abstract

**Introduction:**

The deterioration of social determinants of health across the life course may act as a relevant factor in increasing the risk of frailty and falls, thereby exacerbating social and health problems.

**Objective:**

To analyze whether social disadvantages experienced during different stages of life are associated with the occurrence of falls among older adults.

**Materials and Methods:**

A prospective study was conducted based on the Seniors-ENRICA-1 cohort, including 2,501 Spanish participants aged 60 years or older. Falls and falls requiring medical attention were considered the study outcomes. Exposure was defined as the level of lifelong social disadvantage, assessed across childhood, youth, adulthood, and old age. Bivariate analyses and logistic regression models adjusted for sociodemographic, clinical, and lifestyle variables were applied.

**Results:**

Individuals with low educational attainment were 1.4 times more likely to experience a fall compared to those with higher education. Additionally, individuals without an occupation in adulthood had an 80% higher probability of falling. No significant associations were found with the father's occupation or with household conditions in old age.

**Discussion:**

There is agreement that falls in older adults are associated with accumulated social inequalities, especially low educational attainment and occupation. Variables such as age, lifestyle, and medication use also have an influence. The socioeconomic situation is analyzed from an alternative perspective.

**Conclusion:**

Accumulated disadvantages in education and occupation increase the risk of falls in older adults.

## Resumen

**Introducción:**

El deterioro de los determinantes sociales de la salud a lo largo del curso de vida puede incidir como factor relevante en el aumento del riesgo de fragilidad y caídas agravando los problemas sociales y sanitarios.

**Objetivo:**

Analizar si las desventajas sociales experimentadas durante distintas etapas de la vida se asocian con la ocurrencia de caídas en personas mayores.

**Materiales y métodos:**

Estudio prospectivo basado en la cohorte ENRICA Senior I, con 2.501 participantes españoles de 60 años o más. Se consideraron como desenlaces las caídas y las caídas que requirieron atención médica; y como exposición, el nivel de desventaja social a lo largo de la vida, evaluado en infancia, juventud, adultez y vejez. Se aplicaron análisis bivariados y modelos de regresión logística ajustados por variables sociodemográficas, clínicas y de estilo de vida.

**Resultados:**

Las personas con nivel educativo bajo presentaron 1,4 veces más probabilidad de caerse respecto a quienes tenían estudios superiores; quienes no contaban con ocupación en la adultez mostraron un 80% más de probabilidad de caída. No se hallaron asociaciones significativas con la ocupación del padre ni con las condiciones del hogar en la vejez.

**Discusión:**

Hay concordancia en que las caídas en adultos mayores están asociadas a desigualdades sociales acumuladas, especialmente bajo nivel educativo y la ocupación, variables como edad, estilo de vida y consumo de medicamentos también influyen. La situación socioeconómica cuenta con una perspectiva alternativa de análisis.

**Conclusiones:**

Desventajas acumuladas en educación y la ocupación incrementan el riesgo de caídas en adultos mayores.

## Resumo

**Introdução:**

A deterioração dos determinantes sociais da saúde ao longo da vida pode contribuir significativamente para o aumento do risco de fragilidade e quedas, exacerbando problemas sociais e de saúde.

**Objetivo:**

Analisar se as desvantagens sociais vivenciadas em diferentes fases da vida estão associadas à ocorrência de quedas em idosos.

**Materiais e métodos:**

Estudo prospectivo baseado na coorte ENRICA Senior I, com 2.501 participantes espanhóis com 60 anos ou mais. Os desfechos incluíram quedas e quedas que necessitaram de atendimento médico; a exposição foi definida como o nível de desvantagem social ao longo da vida, avaliado na infância, juventude, idade adulta e velhice. Foram aplicadas bivariadas e modelos de regressão logística ajustados para variáveis sociodemográficas, clínicas e de estilo de vida.

**Resultados:**

Indivíduos com baixa escolaridade apresentaram 1,4 vezes mais probabilidade de sofrer quedas do que aqueles com maior escolaridade; aqueles que estavam desempregados na idade adulta apresentaram uma probabilidade 80% maior de sofrer quedas. Não foram encontradas associações significativas com a ocupação do pai ou com as condições de moradia na velhice.

**Discussão:**

Há consenso de que as quedas em idosos estão associadas a desigualdades sociais acumuladas, especialmente baixa escolaridade e ocupação. Variáveis como idade, estilo de vida e uso de medicamentos também desempenham um papel importante. O status socioeconômico oferece uma perspectiva alternativa para análise.

**Conclusões:**

As desvantagens acumuladas na educação e na ocupação aumentam o risco de quedas em idosos.

## Introduction

Falls are the second leading cause of death from accidental injuries, representing a major global public health problem^1^. Older adults aged 65 years and older are the most affected population^2^,^3^. Ten years ago in the United States, older adults experienced multiple falls each year requiring medical attention, negatively affecting healthcare costs estimated at 28 billion US dollars annually^3^, which are projected to triple due to the growth of this population. However, in Brazil, primary health care annually contributes to reducing the costs associated with falls among older adults, due to the existence of programs such as home health care, which contribute to shortening hospital stays^4^. In Spain, 30% of adults aged 65 years and older experience falls, and half will experience a subsequent fall in the coming months^5^,^6^. These falls will increase over time, with variations in their prevalence associated with functional decline and social and economic conditions.

Similarly, among older adults, the occurrence of falls may be influenced by favorable or unfavorable social and environmental conditions in which they live. These living conditions constitute the theoretical construct and reference known as the social determinants of health, as defined by the World Health Organization, and may have cumulative and differential effects across the life course^7^. This may or may not constitute an advantage in facing the challenges associated with aging^8^. However, the lack of age-appropriate housing conditions and social isolation are considered social disadvantages that increase the risk of falls, and that such an event could result in hospitalization^9^. The differences that exist between socioeconomic levels have a differential impact on some of these groups, increasing the risk of physical disability, frailty, and falls, with their respective social consequences^8^,^10^.

Recently, in France and Sweden, the relationship between falls and multiple factors, including age, frailty, and living environment, was analyzed. Social disadvantages within these social determinants of health have been recently addressed, showing how they negatively interact with dependency among older adults and, collectively, with chronic diseases^11^-^13^. Lifelong social disadvantages and other age-related factors, such as hospitalization in palliative care units^14^, increase the risk of frailty, the risk of falls, and the risk of mortality^14^,^15^, which in 2021 reached a rate of 78 per 100,000 inhabitants^16^. In high-income countries, this situation accounts for between 0.8% and 1.5% of total annual healthcare costs^12^,^17^.

In particular, socially disadvantaged groups due to financial, educational, and employment difficulties are more likely to report frailty and poor health, which could be reversed by improving the living conditions of this population^12^. In this context, social disadvantages in old age are associated with the current social and environmental conditions of the household in which older adults reside and are identified as an important social determinant of health.

The objective of this study was to analyze the influence of lifelong social disadvantages and their association with falls among older adults in Spain.

## Materials and Methods

**Study design**

This cohort study was conducted among adults evaluated in the Seniors Study on Nutrition and Cardiovascular Risk in Spain (ENRICA) 1^18^-^20^. EThe Seniors-ENRICA-1 study was initiated in Madrid, Spain, with a baseline conducted in 2008 among men and women aged 60 and older who were invited to participate voluntarily, and who were followed up on two occasions: 1) 2012-2013 and 2) 2014-2015. The study participants were representative of the Spanish population and were selected using a multi- stage stratified cluster sampling approach. Within each stratum, households were randomly selected, first according to province and municipality size. Households were then selected through telephone dialing, using the telephone directory as the sampling frame. It was possible to estimate prevalence rates with a sampling error of less than 3% and a confidence level of 95%. Likewise, the sample size allowed the detection of associations with relative risks ranging from 1.3 to 1.5, which are considered epidemiologically and clinically relevant.

Households were selected proportionally by sex and age, and sociodemographic data were subsequently collected by telephone. During an in-person visit, blood and urine samples were collected, and physical examinations and dietary-habit interviews were conducted. Overall, the study achieved an above-average response rate of 51%. This figure is comparable to that of other large- scale population studies, which are not free from potential selection bias. However, the stratified random sampling design, together with the application of weighting factors for age, sex, and region, improved the representativeness of the sample and mitigated these limitations.

Participants who had died (n=95) were excluded from the first follow-up in 2012-2013, as were those with evidence of cognitive impairment for whom proxy information (n=18) could not be obtained from their relatives or caregivers. Of the original sample of 2,614 participants^19^, 2,501 older adults formed the final sample and were included in the data analysis.

All study participants provided written informed consent before the assessment began. The study was approved by the clinical research ethics committees of La Paz University Hospital in Madrid and Hospital Clinic in Barcelona^19^.

**Variables and assessment instruments**

**Falls:** These are defined as an involuntary loss of balance and support, which inadvertently causes a person to fall to the ground or to a lower level than where they were. This variable was assessed dichotomously (yes/no) and included in the Seniors-ENRICA-1 study^19^.

**Falls requiring medical attention:** For this category, all events following a fall that resulted in a visit to a physician, attendance at an emergency department, or hospitalization were considered. This variable was measured dichotomously (fall without medical attention/fall with medical attention) and included in the Seniors-ENRICA-1 study^19^.

**Lifelong social disadvantage among older people:** This variable is understood as the combination of different circumstances experienced over the years^21^-^23^. Social disadvantage was assessed across life stages, including childhood, youth, adulthood, and old age; therefore, it was considered that the accumulation of deprivations across these four stages could define lifelong social disadvantage, this variable was measured dichotomously (yes/no) according to whether these characteristics were present Table 1.

**Table 1:** Lifelong social disadvantages throughout the life course: definition

Stage of life	Social disadvantage indicator	Description
Childhood	Father’s occupation	Initial socioeconomic conditions of the household; manual labor^24^ or unskilled occupations.
Youth	Educational level attained	The absence of higher education or completion of only primary/secondary education.
Adulthood	Own occupation	Employment in unskilled, informal jobs or the absence of formal employment.
Old age	Current household conditions	Lack of home ownership, insufficient material resources, or inadequate living conditions.

**Sociodemographic and lifestyle variables:** The following variables were recorded: sex, age, smoking status (never smoker, former smoker, current smoker), alcohol intake (g/day) and/or former drinker (yes/no), adherence to Mediterranean diet (yes/no), dietary calcium intake (continuous), vitamin D intake (continuous), caffeine intake (continuous), physical activity (continuous; leisure time + at home), hours of TV/week (continuous), hours of sleep (continuous; nighttime + daytime), overall obesity (yes/no), abdominal obesity (yes/no), hypertension (yes/no), diabetes (yes/no), cardiovascular disease (yes/no), number of other prevalent diseases (continuous), number of medications (continuous; Medications - 1), use of sleeping pills (yes/no), and history of fracture (yes/ no). These other considerations were taken into account to construct a model aimed at explaining the occurrence of the falls outcome under study.

**Data analysis**

Multinomial logistic regression analysis was conducted using Stata (version 12.0; StataCorp., College Station, Texas, United States). Data distribution normality was assessed using the Shapiro-Wilk test. For quantitative variables, independent-samples t-tests were performed, whereas chi-square tests were used for categorical variables. Odds ratios (ORs) for the risk of falls were estimated for lifelong socioeconomic status, along with their corresponding 95% confidence intervals (95% CI). All p-values < 0.05 were considered statistically significant.

The variables “falls” and “falls requiring medical attention” were analyzed in association with lifelong social disadvantages. Additional analyses were conducted for each variable, including father's occupation, participants' occupation, educational level, and current household disadvantage. Each of these analyses was adjusted for secondary independent variables (confounders) and fitted using three models: Model 1, adjusted for sex and age (continued); model 2, which included model 1 + smoking status (never smoker, former smoker, current smoker), alcohol intake (g/day) and/or former drinker (yes/ no), adherence to Mediterranean diet (yes/no), dietary calcium intake (continuous), vitamin D intake (continuous), caffeine intake (continuous), physical activity (continuous; leisure time + at home), hours of TV/week (continuous), hours of sleep (continuous; nighttime + daytime), overall obesity (yes/no), abdominal obesity (yes/no); and model 3, which included model 2 + hypertension (yes/no), diabetes (yes/no), cardiovascular disease (yes/no), number of other prevalent diseases (continuous), number of medications (continuous; Medications - 1), and use of sleeping pills (yes/no). All collected data is freely available for access and consultation in Mendeley Data^25^.

## Results

Table 2 presents the sociodemographic characteristics, lifestyles, and presence of chronic diseases among older adults evaluated in the Seniors-ENRICA-1 study, according to whether they reported having experienced a fall or not. Additionally, these characteristics are reported for older adults who experienced a fall, distinguishing between those who did not require medical attention and those who did. Significant differences were observed between older adults who reported having experienced a fall and those who reported no falls, and between those who experienced a fall with and without medical attention, in relation to age, tobacco use, alcohol consumption, adherence to the Mediterranean diet, physical activity, presence of cardiovascular disease, and medication use. 

**Table 2:** Characteristics of the older adult population in Spain according to the incidence of falls and falls requiring medical attention. n=2501

Sociodemographic and lifestyle variables	No falls (n=1969) %(n)	Falls (n=532) %(n)	p-value	Falls without medical attention (n=292) %(n)	Falls with medical attention (n=240) %(n)	p-value
Sex						< 0.001*
Men	51.60 (1016)	29.70 (158)	< 0.001*	36.99 (108)	20.83 (50)	
Age. years. Mean ± SD	68.32 ± 6.20	69.91 ± 6.80	< 0.001*	69.23 ± 6.55	70.75 ± 7.02	0.010*
Tobacco use						0.320
Never smoked	55.97 (1102)	69.17 (368)		66.78 (195)	72.08 (173)	
Former smoker	32.00 (630)	22.56 (120)	< 0.001*	25.00 (73)	19.58 (47)	
Current smoker	12.04 (237)	8.27 (44)		8.22 (24)	8.33 (20)	
Alcohol consumption						0.110
Never a drinker	24.33 (479)	35.15 (187)		31.16 (91)	40.00 (96)	
Former drinker	9.60 (189)	10.53 (56)	< 0.001*	9.93 (29)	11.25 (27)	
Moderate consumption	18.33 (361)	19.92 (106)		20.89 (61)	18.75 (45)	
Excessive consumption	47.74 (940)	34.40 (183)		38.01 (111)	30.00 (72)	
Adherence to the Mediterranean diet	24.94 (491)	19.74 (105)	0.013*	23.29 (68)	15.42% (37)	0.020*
Dietary calcium intake. Mean ± SD	890.62 ± 355.74	877.46 ± 330.38	0.440	898.53 ± 340.31	851.84±316.71	0.100
Vitamin D intake. Mean ± SD	3.47 ± 3.17	3.25 ± 2.86	0.140	3.42 ± 2.84	3.04±2.87	0.130
Caffeine intake. Mean ± SD	72.50 ± 108.70	66.59 ± 124.68	0.280	71.38 ± 145.51	60.77±93.27	0.320
Leisure-time PA. MET– h/week. Mean ± SD	22.43 ± 15.57	19.32 ± 13.96	< 0.001*	19.42 ± 14.16	19.19±13.75	0.850
PA at home. MET– h/week. mean ± SD	36.39 ± 32.06	42.36 ± 33.03	< 0.001*	40.18 ± 32.21	45.01±33.89	0.090*
Hours of TV per week. Mean ± SD	17.52 ± 10.62	20.12 ± 13.29	< 0.001*	19.35 ± 13.12	21.05±13.47	0.140
Hours of nighttime sleep. Mean ± SD	6.89 ± 1.35	6.73 ± 1.52	0.010*	6.72 ± 1.41	6.75±1.64	0.780
Hours of daytime sleep. Mean ± SD	0.29 ± 0.55	0.26 ± 0.49	0.240	0.28 ± 0.50	0.23±0.48	0.270
Overall obesity						
Less than 25	20.06 (395)	22.18 (118)		20.21 (59)	0 (0)	2.540
Between 25 and 30	49.06 (966)	45.49 (242)	0.316	44.86 (131)	0.28 (1)	2.540
More than 30	30.88 (608)	32.33 (172)		34.93 (102)	0 (0)	2.540
Abdominal obesity	56.58 (1114)	60.53 (322)	0.100	58.56 (171)	62.92 (151)	0.300
Hypertension	63.59 (1252)	66.17 (352)	0.270	66.78 (195)	65.42 (157)	0.740
Diabetes	15.64 (308)	15.23 (81)	0.810	15.75 (46)	14.58 (35)	0.700
CV disease	2.04 (40)	3.89 (21)	0.020*	93.08 (272)	11.4 (27)	0.360
No. of other prevalent diseases. Mean ± SD	1.64 ± 1.16	2.08 ± 1.34	< 0.001*	1.94 ± 1.32	2.25 ± 1.33	0.009*
No. of medications. Mean ± SD	1.90 ± 1.88	2.31 ± 2.14	<0.001*	2.20 ± 2.08	2.44 ± 2.21	0.200
Use of sleeping pills						
No	82.17 (1618)	70.86 (377)		75.68 (221)	65.00 (156)	
Sometimes	5.89 (116)	6.39 (34)	< 0.001*	6.85 (20)	5.83 (14)	0.015*
Usually	11.93 (235)	22.74 (121)		17.47 (51)	29.17 (70)	

PA=Physical activity, CV=Cardiovascular, h=hours, SD=Standard deviation, MET= metabolic equivalent of task (energy cost of a person at rest). No.=Number. For quantitative variables, independent-samples t-tests were performed, and for categorical variables, a chi-square tests were used. *Statistically significant differences were considered at p<0.05.

No differences were observed in lifelong social disadvantages between older adults who reported having experienced a fall and those who reported no falls, nor between those who experienced a fall with or without medical attention Table 3. 

**Table 3:** Lifelong social disadvantage of older adults according to fall risk. n=2,501.

Lifelong social disad- vantage of older adults	No falls (n=1969) %(n)	With falls (n=532 ) %(n)	p-value	Falls without medical attention (n=292) %(n)	Falls with medical attention (n=240) %(n)	p-value
Disadvantage in child-hood						0.380
Father's occupation: manual labor	33.01 (650)	31.39 (167)	0.470	29.79 (87)	33.33 (80)	
Disadvantage in youth: Level of education						0.110
Primary education	53.89 (1061)	56.95 (303)		53.08 (155)	61.67 (148)	
Secondary education	24.53 (483)	23.68 (126)	0.400	25.00 (73)	22.08 (53)	
University education	21.58 (425)	19.36 (103)		21.92 (64)	16.25 (39)	
Disadvantage in adulthood						0.340
Own occupation: manual labor	28.34 (558)	34.21 (182)	0.008	35.96 (105)	32.08 (77)	
Disadvantage in old age - Current household conditions						0.610
At least one	83.44 (1643)	85.15 (453)	0.340	82.53 (241)	89.33 (214)	
Lifelong social disadvantage						0.670
All	30.27 (596)	27.63 (147)	0.620	31.16 (91)	23.33 (56)	
Continuing lifelong social disadvantage. Mean ± SD	2.17 ± 0.72	2.15 ± 0.72	0.560	2.12 ± 0.73	2.18 ± 0.72	0.340

For quantitative variables, independent-samples t-tests were performed, and for categorical variables, a chi-square tests were used. * Statistically significant differences were considered at p<0.05.

**Figure 1:**
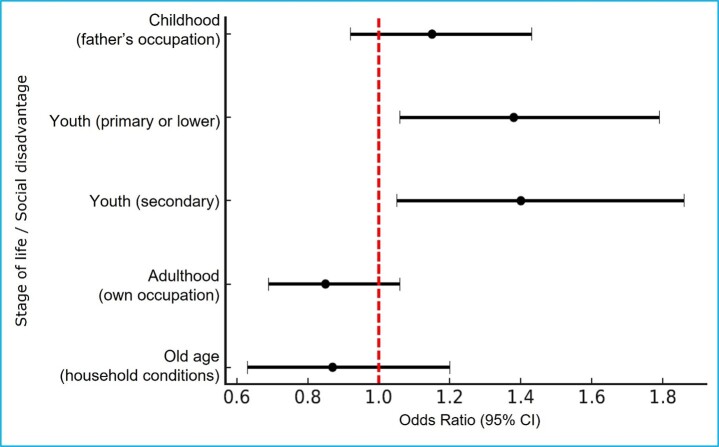
Lifelong social disadvantage throughout life and risk of falls in older adults

Table 4 and Figure 1 show the association between lifelong social disadvantage and falls among older adults. In the model adjusted for sociodemographic characteristics, lifestyles, and the presence of chronic diseases (Model 3), older adults with primary or lower education and those with secondary education had a 1.4-fold higher likelihood of experiencing a fall than those with higher education. 

Furthermore, in the model adjusted for sex and age, older adults without an occupation in adulthood had an 80% higher likelihood of experiencing a fall compared with those who had their own occupation. Regarding social disadvantages assessed in childhood (father's occupation) and in old age (current household conditions), no significant associations were observed. 

**Table 4:** Association between lifelong social disadvantage and falls in older adults in Spain

Variables	Model 1	p-value	Model 2	p-value	Model 3	p-value
	OR ( 95% CI)		OR ( 95% CI)		OR ( 95% CI)	
**Childhood (Father's occupation)**						
Without disadvantage	1		1		1	
Disadvantage	1.05 (0.85-1.30)	0.639	1.08 (0.87-1.34)	0.467	1.15 (0.92-1.43)	0.216
**Youth (Own level of education)**						
Higher Education	1		1		1	
Primary or lower education	1.15 (0.90-1.47)	0.261	1.27 (0.99-1.64)	0.062	1.38 (1.06-1.79)	0.015*
Secondary education	1.13 (0.87-1.47)	0.351	1.32 (1.00-1.74)	0.053	1.40 (1.05-1.86)	0.022*
**Adulthood (Own occupation)**						
Without disadvantage	1		1		1	
Disadvantage	0.80 (0.63-0.96)	0.019*	0.82 (0.66-1.01)	0.063	0.85 (0.69-1.06)	0.159
**Old age (Current household conditions)**						
Without disadvantage	1		1		1	
Disadvantage	0.98 (0.72-1.34)	0.903	0.90 (0.65-1.24)	0.512	0.87 (0.63-1.20)	0.385

Multinomial logistic regression analysis was performed. Odds ratios (ORs) of falls in relation to lifelong socioeconomic status were estimated, along with their corresponding 95% confidence intervals (95% CI). Model 1: adjusted for sex and age (continuous). Model 2: Additionally adjusted for smoking status (never smoker, former smoker, current smoker), alcohol intake (never drinker, former drinker, moderate alcohol intake, excessive alcohol intake), adherence to Mediterranean diet (yes/no), dietary calcium intake (continuous), vitamin D intake (continuous), caffeine intake (continuous), leisure-time physical activity (continuous), physical activity at home (continuous), hours of TV/ week (continuous), hours of nighttime sleep (continuous), hours of daytime sleep (continuous), overall obesity (yes/no), abdominal obesity (yes/no). Model 3: additionally adjusted for hypertension (yes/no), diabetes (yes/no), cardiovascular disease (yes/no), number of other prevalent diseases (continuous), number of medications (continuous), and use of sleeping pills (yes/no). *p<0,05

*The forest plot shows the odds ratios (ORs) and their 95% confidence intervals for the relationship between social disadvantage across different life-course stages (childhood, youth, adulthood, and old age) and the risk of falls among older adults. The values were adjusted according to the multivariable Model 3, considering sociodemographic and health variables. The red dotted vertical line indicates the reference value (OR= 1). Points to the right of the line indicate a higher risk of falls, whereas points to the left indicate a lower risk of falls.*

**Figure 2:**
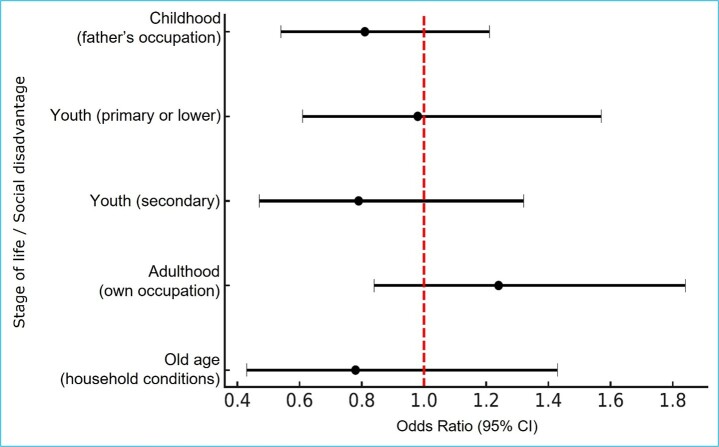
Lifelong social disadvantage and risk of falls requiring medical attention in older adults

Additionally, no significant association was observed between lifelong social disadvantage and falls requiring medical attention Table 5 and Figure 2.

**Table 5:** Association between lifelong social disadvantage and falls in older adults in Spain

Variables	Model 1	p-value	Model 2	p-value	Model 3	p-value
	OR ( 95% CI)		OR ( 95% CI)		OR ( 95% CI)	
**Childhood (Father's occupation)**						
Without disadvantage	1		1		1	
Disadvantage	0.80 (0.55-1.17)	0.244	0.78 (0.53-1.16)	0.222	0.81 (0.54-1.21)	0.305
**Youth (Own level of education)**						
Higher Education	1		1		1	
Primary or lower education	0.92 (0.60-1.43)	0.726	0.78 (0.60-1.50)	0.808	0.98 (0.61-1.57)	0.934
Secondary education	0.84 (0.52-1.36)	0.477	0.78 (0.47-1.30)	0.334	0.79 (0.47-1.32)	0.368
**Adulthood (Own occupation)**						
Without disadvantage	1		1		1	
Disadvantage	1.29 (0.88-1.86)	0.192	1.21 (0.82-1.77)	0.339	1.24 (0.84-1.84)	0.284
**Old age (Current household conditions)**						
Without disadvantage	1		1		1	
Disadvantage	0.82 (0.47-1.42)	0.471	0.86 (0.48-1.52)	0.600	0.78 (0.43-1.43)	0.431

Odds ratios (ORs) for falls in relation to lifelong socioeconomic status were estimated, along with their corresponding 95% confidence inter- vals (95% CI). Model 1: adjusted for sex and age (continuous). Model 2: Additionally adjusted for smoking status (never smoker, former smoker, current smoker), alcohol intake (never drinker, former drinker, moderate alcohol intake, excessive alcohol intake), adherence to Mediterranean diet (yes/no), dietary calcium intake (continuous), vitamin D intake (continuous), caffeine intake (continuous), leisure-time physical activity (continuous), physical activity at home (continuous), hours of TV/week (continuous), hours of nighttime sleep (continuous), hours of daytime sleep (continuous), overall obesity (yes/no), abdominal obesity (yes/no). Model 3: additionally adjusted for hypertension (yes/no), diabetes (yes/no), cardiovascular disease (yes/no), number of other prevalent diseases (continuous), number of medications (continuous), and use of sleeping pills (yes/no). *p<0,05.

*The forest plot presents odds ratios (ORs) and their 95% confidence intervals for the association between different indicators of lifelong social disadvantage and the occurrence of falls requiring medical attention among older adults. The estimates correspond to multivariable Model 3. The red dotted vertical line indicates the reference value (OR = 1). Values greater than 1 reflect a higher probability of falls requiring medical attention, whereas values less than 1 suggest a lower probability.*

## Discussion

The results of this study reveal inequalities in falls among older adults in Spain. A higher probability of falls was observed among older adults with lower levels of education and without their own occupation.

Being female, older age, and behavioral factors such as watching television increase the risk of falls. Additionally, biological factors such as lifestyle, diabetes, and prevalent diseases, as well as the number of medications taken and the use of sleeping pills, were associated with falls in this study. No clear association was observed for socioeconomic status, specifically within categories that included occupation type.

It has been shown that lifelong socioeconomic inequalities affect health capital in old age. Regardless of how these inequalities are measured, the outcome is the same. Our study presents socioeconomic levels estimated across four life periods, in a manner similar to that described by Herr et al.^12^ However, in our study, childhood socioeconomic status was characterized based on the father's occupation, whereas in the French study, living standards were considered for this criterion. Furthermore, those researchers assessed the financial situation as an indicator of socioeconomic position in the most advanced stage of life, whereas in our study, household conditions were used for this phase.

Deprivation estimates reported in the studies by Khow et al.^26^ and Enderlin et al.^27^ qassociate the risk of falls in older adults with poor lighting, slippery surfaces, clutter, rugs, lack of ramps, lack of seat-lift devices, and lack of handrails. These findings are consistent with the deprivation analysis in our study, which identified conditions such as lack of heating, reporting feeling cold, and absence of an elevator.

Our study shows that economic, social, occupational, and educational conditions accumulated throughout life influence the risk of falls in older adults. Factors such as access to quality education and dignified work function as protective factors, whereas individuals who experience structural disadvantages in these areas are influenced by the economic and political models of each territory. In line with Lander, these inequalities are situated within a civilizational crisis in which capital, by prioritizing individual profit and corporate interests, deepens social and ecological asymmetries, thereby weakening solidarity and democracy. This highlights the need to engage in discussions on public social protection policies that recognize the impact of these demographic and social changes, adapt to population aging, and promote dignified, equitable, and solidarity-based aging in the context of a system that transfers the burden of care to families^28^.

Our results show an association between falls and increasing age. This result is consistent with those reported by Morrison et al.^29^, who indicate that the increased risk of falls is associated with a decline in physiological processes related to balance and gait. According to studies by Mata et al^30^. low levels of education are associated with frailty and falls. These results are consistent with our results, in which these outcomes were less frequent among participants with university-level or higher education low levels of education are associated with frailty and falls. These results are consistent with our results, in which these outcomes were less frequent among participants with university-level or higher education.

Furthermore, in our study, the use of sleeping pills, considered as a secondary independent variable, was associated with falls among older adults in Spain. Similarly, independent studies conducted in different settings by Martinez et al.^31^, como también por el grupo de Seitz et al.^32^ and by Diaz et al.^33^ have reported the same result.

This research provides a systematic assessment of the association between the level of social disadvantage, specifically in relation to household conditions, and falls, showing that the risk of falls increases as the level of education decreases.

Our research did not include institutionalized subjects, a circumstance in which adults are more prone to falling; therefore, the risk in this population group is underestimated. As in other population-based surveys, selection bias may be present, as approximately 25% of participants did not take part in the follow-up; consequently, information from these participants was not included in this study in order to address this situation.

One implication of our results is the need for government involvement in the implementation of interventions aimed at reducing the incidence of falls. Governments can promote public policies related to the design and construction of neighborhoods whose standards and conditions enable older adults to maintain mobility, through age-friendly urban design,^34^ cand through housing and environments equipped with devices to support older adults with mobility limitations.

A response rate of 51% may represent a limitation of this study. Although this value is comparable to that reported in other large population-based studies, it does not preclude the presence of potential selection bias. Therefore, the use of a stratified random sampling design strengthened the representativeness of the sample by applying weighting factors for age, sex, and region, thereby mitigating this limitation. Additionally, another limitation was that, although the study focuses on the general population of non-institutionalized older adults, individuals with severe cognitive impairment, serious communication difficulties, or conditions that prevented participation in the interview were excluded. This exclusion may have introduced selection bias, as older adults with greater social and clinical vulnerability were left out.

## Conclusions

This study analyzed the association between social disadvantages and falls among older adults in Spain from a multifactorial relationship^35^, It should be emphasized that these conditions of inequality act as factors that contribute to and increase the risk of falls. Among the social disadvantages analyzed, not all showed significant associations; however, among those with the strongest associations, educational level and individuals’ own occupation were identified. Given that falls involve a set of interacting dimensions, this study presents a challenge to continue analyzing other factors that, although not statistically significant, such as social disadvantage in childhood and old age, may also contribute to fall incidents. For this reason, identifying these factors is relevant for proposing strategies to mitigate fall risk factors to prevent these outcomes among older adults^36^,^37^.
